# The Practice of Pancreatoduodenectomy in India: A Nation-Wide Survey

**DOI:** 10.7759/cureus.41828

**Published:** 2023-07-13

**Authors:** Gourav Kaushal, Nirjhar Raj Rakesh, Anvin Mathew, Sumit Sanyal, Abhishek Agrawal, Puneet Dhar

**Affiliations:** 1 Surgical Gastroenterology, All India Institute of Medical Sciences, Bathinda, Bathinda, IND; 2 Surgical Gastroenterology, All India Institute of Medical Sciences, Rishikesh, Rishikesh, IND; 3 Surgical Gastroenterology, Narayana Multispeciality Hospital, Kolkata, IND; 4 Surgical Gastroenterology, Amrita School of Medicine, Faridabad, IND

**Keywords:** indian association of surgical oncology, indian association of surgical gastroenterology, pancreatic cancer, pancreas, hepato-pancreato-biliary surgery, pancreatoduodenectomy, whipples operation, carcinoma pancreas

## Abstract

Introduction

The way pancreatoduodenectomy (PD) is performed can vary a lot around the world, and there is no agreed-upon standard approach. To learn more about how PD is practised in India, a survey was conducted among Indian surgeons to gather information about their current practices.

Methods

A survey was created and shared with surgeons in India who practice pancreatic surgery. It had 33 questions that aimed to capture information about different aspects of PD practice. These questions covered topics such as the surgeons' education and experience, how they evaluated patients before surgery, what they considered during the operation, and how they managed patients after surgery.

Results

A total of 129 surgeons were sent the survey, and 110 of them completed it. The results showed that 40.9% of the surgeons had less than five years of experience, and 36.4% of them performed more than 15 PDs in a year. When deciding whether to perform preoperative biliary drainage, 60% of surgeons based their decision on the level of bilirubin in the patient's blood, while the rest considered other specific indications. The majority of surgeons (72.7%) looked at the trend of albumin levels to assess the patient's nutritional status before surgery. Venous infiltration was seen as a reason for neoadjuvant therapy by 76.4% of the participants, whereas 95.5% considered upfront surgery in cases of venous abutment. When it came to the type of PD, 40% preferred classical PD, 40.9% preferred pylorus-resecting PD (PRPD), and the rest chose pylorus-preserving PD (PPPD). Pancreatojejunostomy (PJ) was the preferred method for 77.3% of surgeons, while 6.3% preferred pancreatogastrostomy (PG). About 65.5% of surgeons used octreotide selectively during the operation when the duct diameter was small. Nearly all surgeons (94.5%) preferred to secure feeding access during PD, and all of them placed intraperitoneal drains. As for postoperative care, 37.3% of surgeons attempted early oral feeding within 48 hours, while 28.2% preferred to wait at least 48 hours before initiating oral feeds.

Conclusions

The survey revealed significant differences in how PD is practised among surgeons in India, highlighting the heterogeneity in their approaches and preferences.

## Introduction

According to the GLOBOCAN 2018 estimates, pancreatic cancer is the 11th most common cancer worldwide, accounting for approximately 4.5% of all cancer-related deaths [1]. Pancreatoduodenectomy (PD) is the main surgical procedure used for curative treatment. However, PD is a technically challenging operation and experienced centres still observe a postoperative mortality rate of 3-5%. Additionally, post-surgery complications remain high, ranging from 30-61%. Evidence suggests that specialized units with high surgical volumes have better outcomes, with operative mortality rates below 5% [2]. Therefore, there is a push to centralize and standardize complex surgical procedures like PD.

There is significant variation in the way PD is practiced worldwide and an ongoing debate about the best techniques for resection and reconstruction [2-5]. While there is no universally agreed-upon standard, efforts are being made to establish standardized guidelines for evaluating and performing PD in order to achieve optimal results.

Considering this premise, a survey was conducted among Indian surgeons to acquire comprehensive data pertaining to the current practice of PD. This survey serves as a pivotal preliminary measure in the endeavor to establish a standardized practice in this part of the world.

## Materials and methods

A web-based survey was created and administered to surgeons who specialize in pancreatic surgery in January-March, 2021. The survey was distributed through e-mail by the investigators to potential participants identified through professional associations such as the Indian Association of Surgical Gastroenterology, the Indian Association of Surgical Oncology, and the International Hepato-Pancreato-Biliary Association-Indian Chapter. However, while participants were invited from most of the states of the country, there is no exact data on which region each participant belonged to. To ensure a representative sample from various regions of India, a snowball sampling method was employed, where participants were asked to recommend potential future participants. If potential participants failed to complete the questionnaire, two follow-up attempts were made to elicit participation through electronic correspondence. The survey was administered exclusively in English.

The survey instrument (see Appendix) consisted of 33 objective items, which were designed to capture information on various aspects of PD practice, including the educational qualifications and experience of surgeons, preoperative evaluation of patients requiring PD, operative considerations, and postoperative management. By assessing these four domains, the study aimed to provide a comprehensive understanding of the practice of PD in India.

In the domain of training and experience, the survey sought to elicit information on the highest level of education attained by the surgeon, the number of years of experience in surgical practice, and the average annual volume of PDs performed. This information was used to characterize the respondent's current clinical practice. In the domain of preoperative evaluation, the survey enquired about the surgeon's philosophy regarding preoperative biliary drainage, the assessment of preoperative nutritional status, and the decision-making process for upfront surgery. In the domain of operative aspects, the survey sought information on the surgical approach used (open or minimally invasive), the level of transection of the proximal gastrointestinal tract, the method for pancreas parenchymal transection, the technique for pancreatic-luminal anastomosis, the methods used to prevent postoperative pancreatic fistula (POPF), and the use of drains and feeding access. In the domain of postoperative management, the survey enquired about the timing of initiation of feeding, the timing of removal of the nasogastric tube (NG), and the timing of removal of the drain, with a specific focus on POPF. All questions were objective in nature, with four or five options provided for each question.

## Results

Demographic and experience details

**Figure 1:**
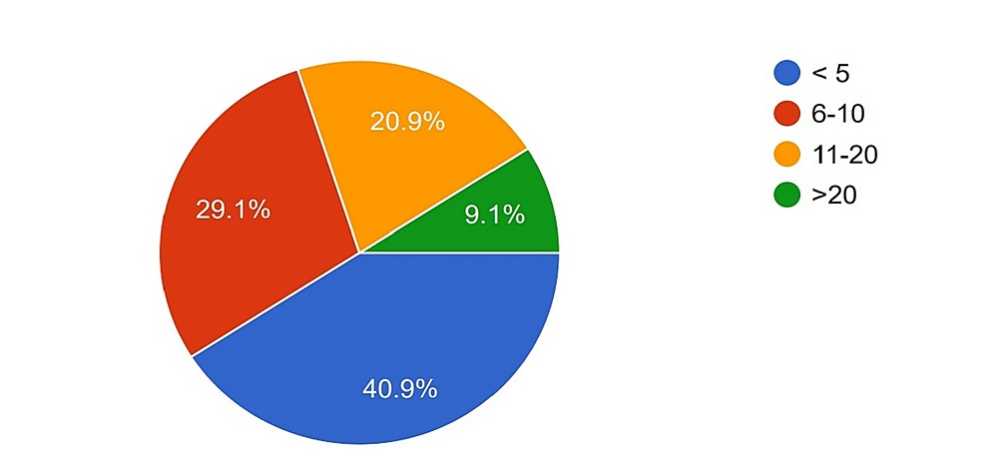
Experience of the surgeons in number of years after obtaining the highest degree. <5: Less than five years of experience; 6-10: 6-10 years of experience; 11-20: 11-20 years of experience; >20: More than 20 years of experience

**Figure 2:**
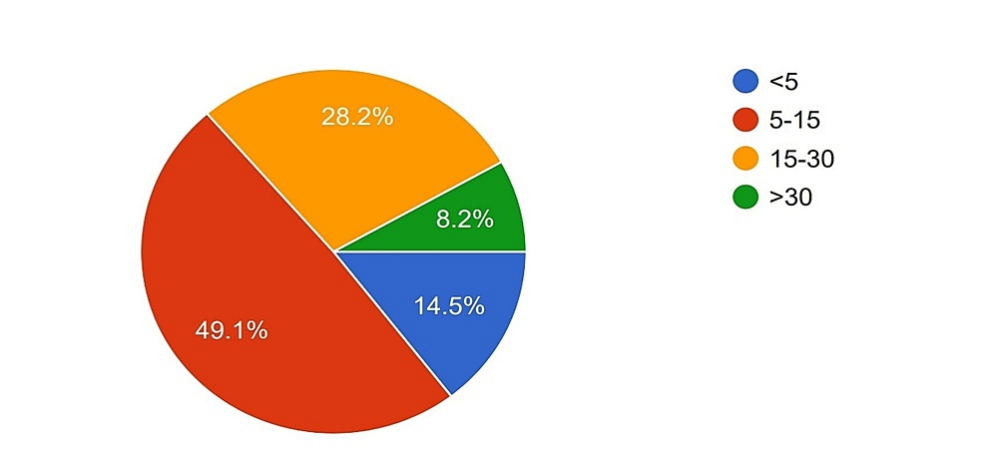
Average number of pancreaticoduodenectomies done by the surgeons per year. <5: Less than five surgeries/year; 5-15: 5-15 surgeries/year; 15-30: 15-30 surgeries/year; >30: More than 30 surgeries/year

A total of 129 participants were sent the questionnaire, of which 110 completed it. The respondents' ages ranged from 31 to 70 years, with a median age of 40. All participants were male. Regarding the training received, 90 participants (81.8%) had subspecialty training in the form of MCh (Master of Chirurgiae (surgery))/DNB (Diplomate of National Board), which is three-year higher specialty training in India, in Gastrointestinal Surgery or Surgical Oncology, while 20 (18.2%) had General Surgery training along with fellowship training in Hepato-Pancreato-Biliary Surgery, Organ Transplant Surgery, etc. Most participants were in the early phase of their surgical practice, with 40.9% having less than five years of experience after obtaining their highest degree, 29.1% having 6-10 years of experience, and 20.9% having 11-20 years of experience. Only 9.1% of the participants had more than 20 years of experience (Figure 1). In terms of the number of PDs performed per year, nine surgeons (8.2%) reported performing more than 30 PDs, 31 (28.2%) reported performing 15-30 PDs, and 49.1% reported performing 5-15 PDs. Fifteen (8.2%) surgeons reported performing less than five PDs per year (Figure 2).

Preoperative considerations

Preoperative Drainage

**Figure 3:**
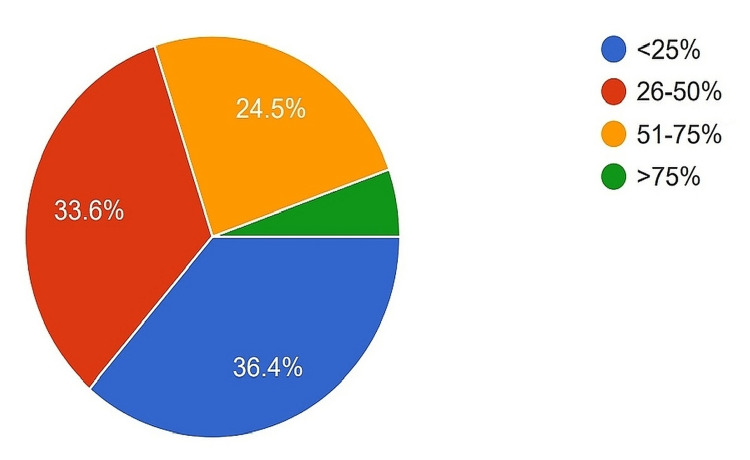
Proportion of patients referred to each surgeon after CBD stenting <25% : Surgeon receives <25% of the patients stented; 26-50%:  Surgeon receives 25-50% of the patients stented; 51-75%:  Surgeon receives 51-75% of the patients stented; >76%:  Surgeon receives 51-75% of the patients stented CBD: common bile duct

Thirty percent of the surgeons reported that they receive more than 50% of the referred patients with stents in situ (Figure 3). When it came to the philosophy of preoperative drainage, 40% of the surgeons believed that serum bilirubin levels greater than 15 g/dl, and 16.4% believed that levels greater than 20 g/dl were an indication for preoperative drainage. However, 40% of the surgeons did not consider preoperative drainage based solely on serum bilirubin levels unless complications, such as cholangitis, were present.

Preoperative Nutrition

**Figure 4:**
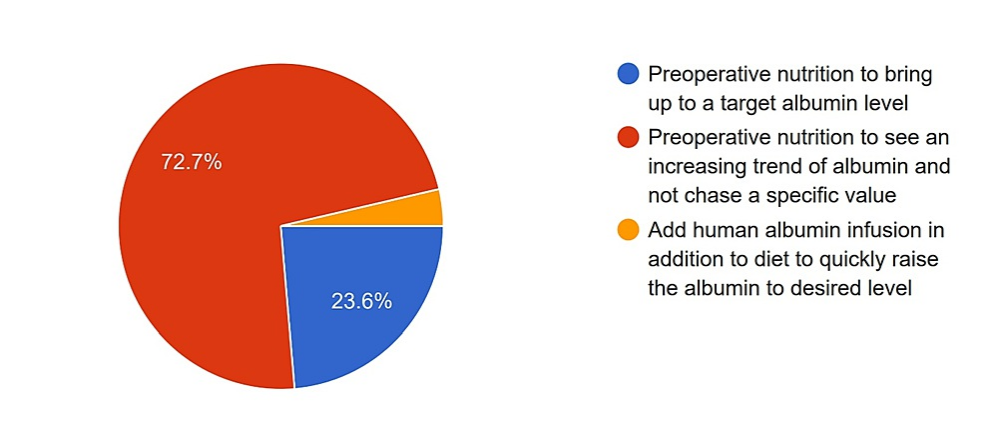
Strategies adopted by the surgeons to improve the nutritional status in a patient with poor nutritional status.

Regarding preoperative nutritional status, 72.7% of the respondents noted that they would look for an increasing trend in serum albumin levels instead of a specific target value when determining fitness for surgery. In contrast, 26 (23.6%) respondents preferred to use a specific target value of serum albumin to assess fitness for surgery. Only four (3.7%) surgeons believed that preoperative albumin infusion could be used to address poor preoperative status (Figure 4).

Upfront Surgery

**Figure 5:**
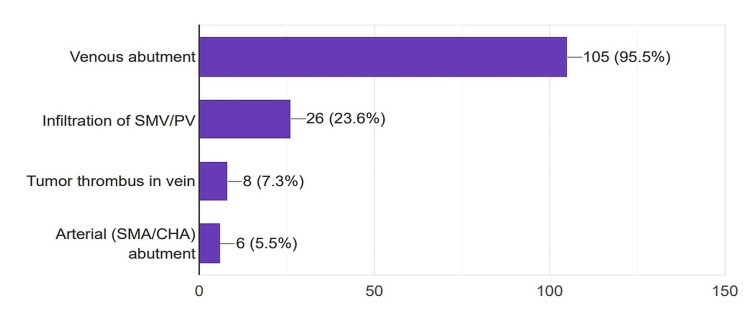
Indications for an upfront surgery considered by the surgeons. SMV: Superior mesenteric vein; PV: Portal vein, SMA: Superior mesenteric artery; CHA: Common hepatic artery

Regarding upfront surgery, 95.5% of the surgeons preferred to proceed with an upfront surgery in cases of venous (superior mesenteric vein-portal vein) abutment of the tumor. However, 76.4% (84) of the surgeons tended to offer neoadjuvant therapy before surgery in cases of venous infiltration. Interestingly, 5.5% (six) of the participants considered upfront surgery even in cases of suspected arterial involvement (Figure 5).

Intraoperative considerations

Surgical Approach

**Figure 6:**
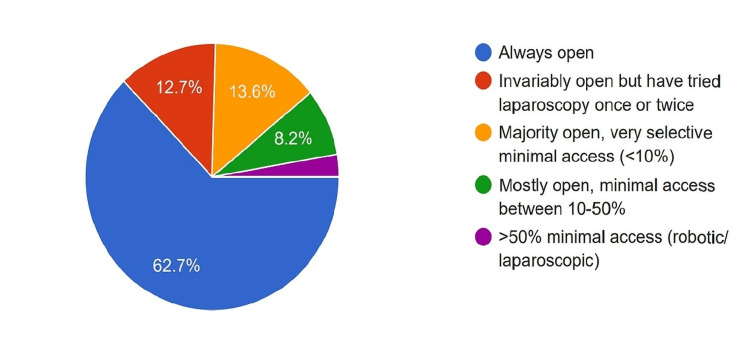
Adoption of minimally invasive pancreatoduodenectomy among the surgeons.

In the survey, it was found that the majority (62.7%) performed open PD, while 13.6% selectively performed minimally invasive PD (<10% of surgeries). A small percentage (8.2%) performed more than 10% of PDs minimally invasive, and only 2.8% performed more than 50% of PDs in this way (Figure 6).

Level of Transection of Proximal GastrointestinaI Tract

**Figure 7:**
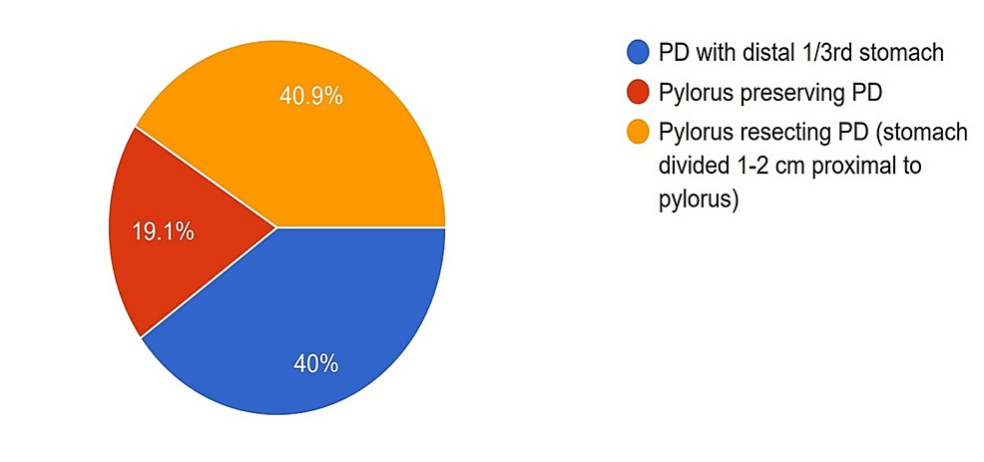
Level of proximal GI tract division preferred by the surgeons. PD: Pancreatoduodenectomy

Forty percent of the surgeons performed classical PD where the distal one-third of the stomach is removed; meanwhile, 40.9% of surgeons performed pylorus-resecting PD (PRPD) where the stomach is transected 1-2 cm proximal to the pylorus. Only 19.1% of the participants prefer to perform pylorus-preserving PD (PPPD) (Figure 7). Regarding the philosophy behind the level of gastric resection, 40.9% of the participants believed that pylorus preservation might lead to delayed gastric emptying, 24.5% believed that the extent of gastric resection was linked to postoperative malnutrition, and 15.5% believed that a greater extent of gastric resection lead to better lymphadenectomy.

Lymphadenectomy

**Figure 8:**
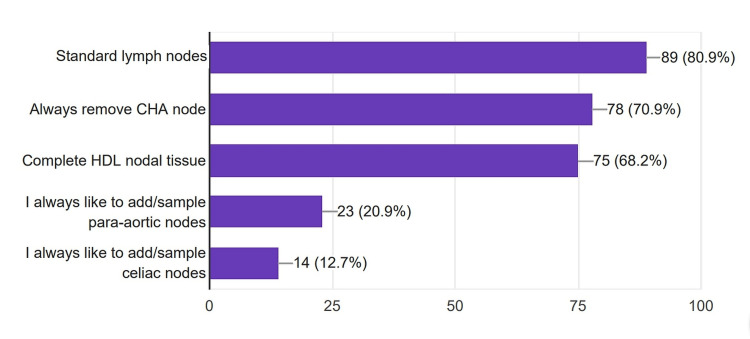
Extent of lymphadenectomy done by the surgeons. CHA: Common hepatic artery; HDL: Hepato-duodenal ligament

Regarding lymphadenectomy, 70.9% of the surgeons removed common hepatic artery station nodes, and 68.2% did hepatoduodenal lymphadenectomy along with the nodes that are removed with the specimen (Figure 8).

Pancreas Transection

The method of pancreatic transection varied, with 55.5% of the surgeons using electrocautery, 25.5% using energy devices, 13.6% using cold knives, and the remaining using a combination of methods with personalized modifications.

Pancreatico-enteric Anastomosis

**Figure 9:**
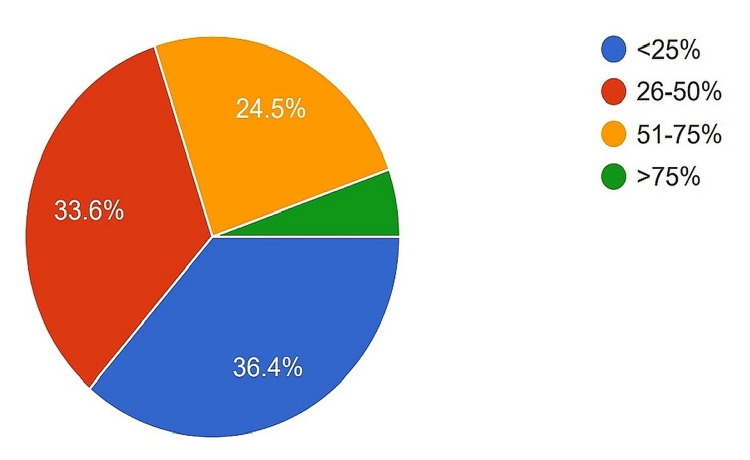
Preferred organ for pancreatic remnant drainage as reported by the surgeons. PG: Pancreaticogastrostomy; PJ: Pancreatojejunostomy

Most surgeons (77.3%) preferred to construct a pancreatojejunostomy (PJ) routinely to drain the remnant pancreas, while 6.3% preferred a pancreaticogastrostomy (PG) and 15.5% a tailor-made approach. Only one surgeon used an isolated Roux-en-Y jejunal limb for PJ construction (Figure 9). Regarding anastomosis techniques, 71.8% of surgeons constructed a PJ using the duct-mucosa technique, and 24.6% used invagination techniques; 27.3% of the surgeons used a magnification device such as a loupe, either always or selectively.

Trans-anastomotic Stents

**Figure 10:**
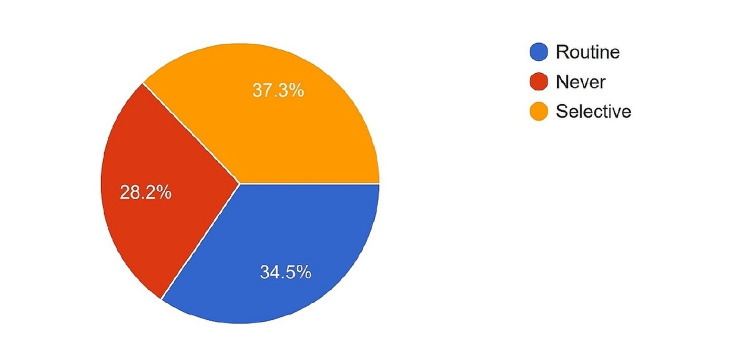
Proportion of the surgeons using trans-anastomotic stents across pancreatic duct anastomosis.

Of the surgeons, 34.5% used trans-anastomotic stents in all pancreatic-digestive anastomosis, while another third used them selectively, and 28.2% did not use stents. Among the surgeons who used a stent, 86.1% drained it internally (Figure 10).

Perioperative Octreotide

**Figure 11:**
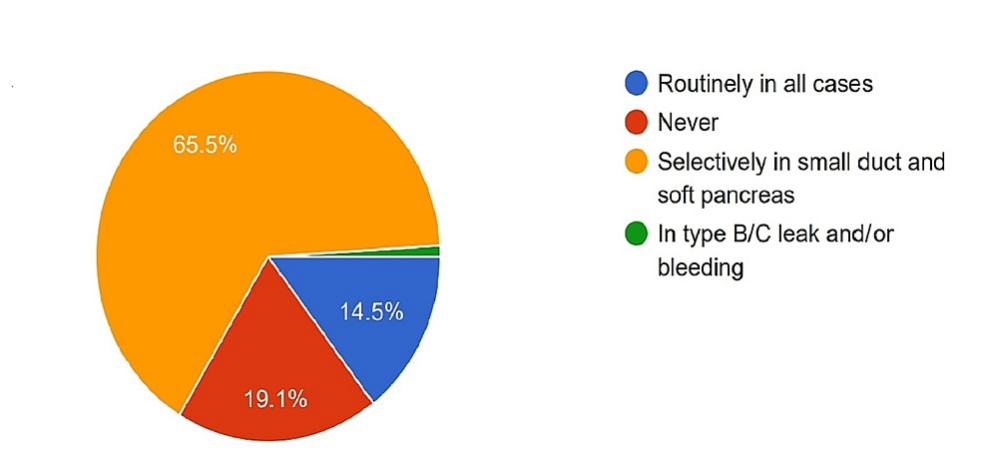
Proportion of the surgeons using perioperative octreotide or its analogue.

Perioperative octreotide was used by 14.5% of surgeons to prevent POPF, while 19.1% did not use it at all. The majority (65.5%) preferred to use it selectively in the case of a soft pancreas or small diameter duct (Figure 11).

Biliary-enteric Anastomosis

**Figure 12:**
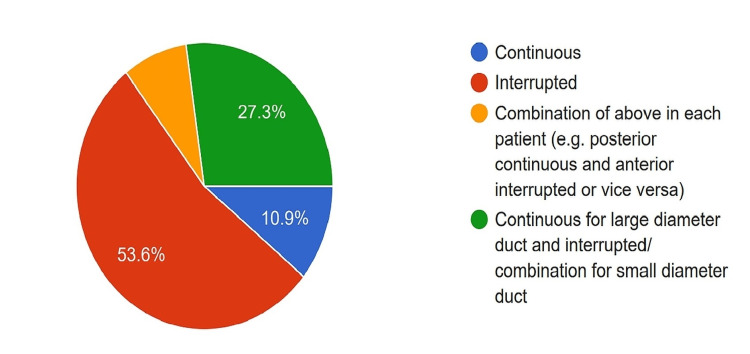
Proportion of surgeons using various techniques of biliary-enteric anastomosis.

Regarding biliary enteric anastomosis, 53.6% of surgeons used interrupted sutures, 10.9% used continuous sutures irrespective of bile duct diameter, and 27.3% used a tailor-made approach based on bile duct diameter (Figure 12).

Feeding Access

**Figure 13:**
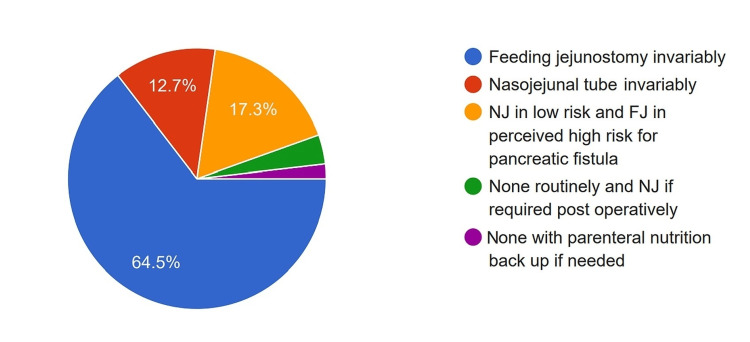
Pie diagram showing proportion of surgeons using various routes for administering enteral feeds. FJ: Feeding jejunostomy, NJ: Naso-Jejunal tube

Nearly all surgeons (94.5%) preferred to secure feeding access when performing a PD, with 64.5% preferring a feeding jejunostomy (FJ) and 12.7% using a nasojejunal tube (NJ). In comparison, 17.3% used an FJ or NJ, depending on the risk of developing a POPF (Figure 13).

Peritoneal Drains

All surgeons preferred to place intraperitoneal drains; 36.4% of surgeons used two drains, one near the PJ site and another in the subhepatic space, 31.8% used two drains selectively, and 29.1% preferred to use a single drain.

Duration of Surgery

The duration of the surgery varied, with 30% of surgeons claiming to complete a standard resection and reconstruction (no vascular resection and standard lymphadenectomy) in less than five hours, while 65.5% took five to eight hours.

Postoperative considerations

Start of Oral Feeds

**Figure 14:**
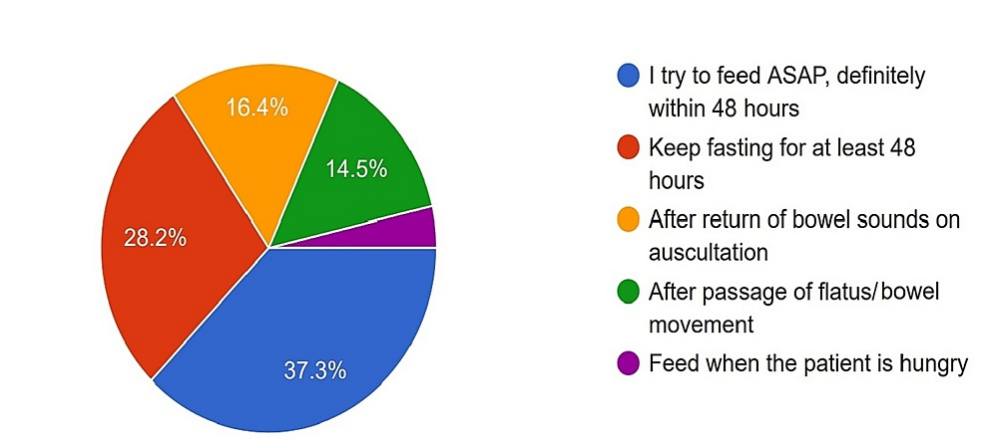
Preference of the surgeons while starting oral feeds. ASAP: As soon as possible

Of the surgeons, 37.3% initiated early oral feeding within 48 hours, while 28.2% preferred to maintain fasting for at least 48 hours; 16.4% of surgeons based their decision on the return of bowel sounds, while 14.5% waited for the patient to pass flatus or stool before starting oral feeds (Figure 14).

Start of Enteral Feeds

**Figure 15:**
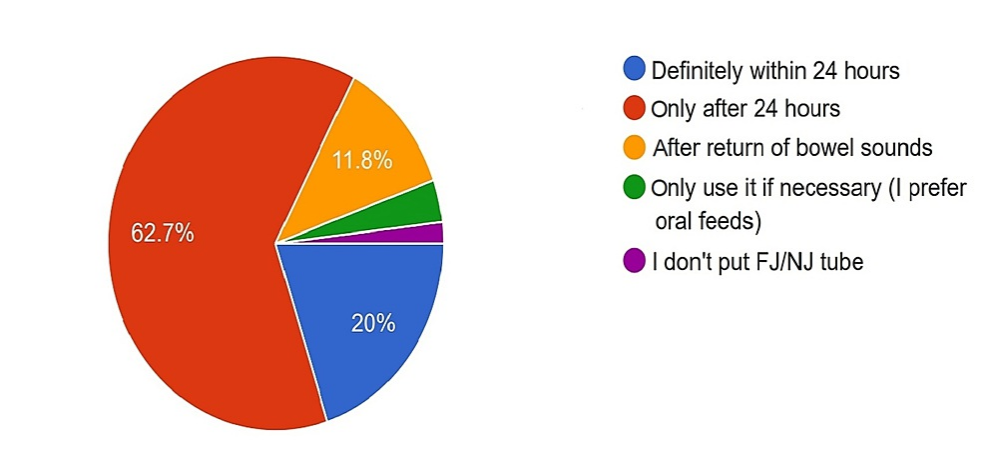
Preference of surgeons in starting enteral feeds. FJ: Feeding jejunostomy; NJ: Nasojejunal tube

With regard to enteral feeds, 20% of surgeons initiated them within 24 hours, 62.7% initiated them after 24 hours, and 11.8% waited for the return of bowel sounds (Figure 15).

Intravenous Fluids

Ringer lactate and normal saline were the preferred fluids in the postoperative period, with 55.5% of the surgeons choosing each, while 51.7% chose dextrose-containing solutions such as dextrose 5% (D5) or dextrose normal saline (DNS); 15.5% of the surgeons used albumin infusion, and 6.4% used colloids.

Monitoring for POPF

**Figure 16:**
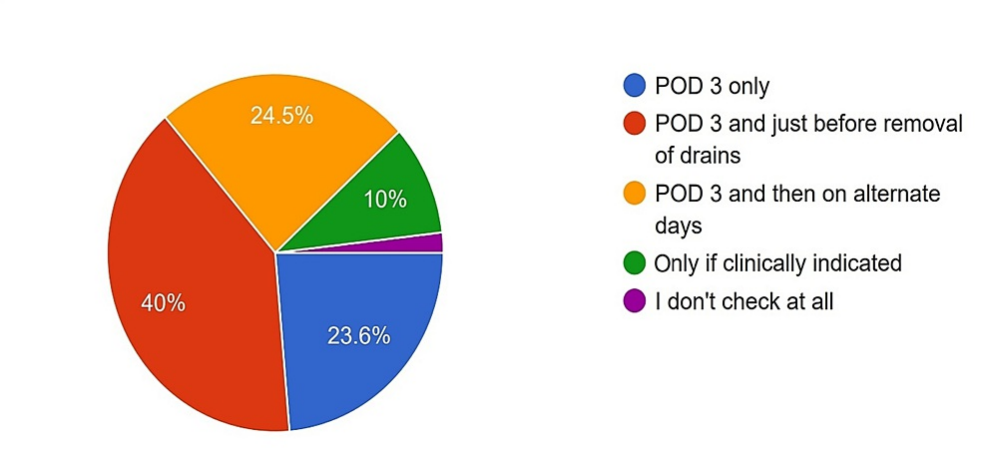
Preference of the surgeons in testing drain fluid amylase levels to look for postoperative pancreatic fistula. POD: Postoperative day

Regarding monitoring for POPF, 40% of the surgeons checked drain fluid amylase levels on postoperative day three and before drain removal, 24.5% checked them on alternate days starting from postoperative day three until drain removal, 23.6% checked them only on postoperative day three, 10% checked them only when clinically indicated, and 1.9% did not rely on this method at all (Figure 16).

Removal of Drain

**Figure 17:**
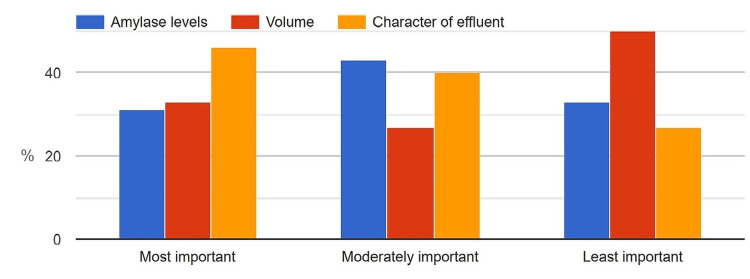
Key characteristics of drain effluent influencing the surgeons' decision on drain removal.

More than 40% of the surgeons (42.7%) considered the character of the effluent as the most critical factor determining drain removal; meanwhile, 29% considered the most critical factor to be drain amylase levels and 28.3% believed it to be the volume of the effluent (Figure 17).

Duration of Hospital Stay

The majority of the surgeons (62.7%) advised a stay of 8-10 days. 26.5% of the surgeons suggested a stay of five to seven days, while a small percentage (9.1%) advised a stay of 11-15 days during preoperative counseling.

## Discussion

This survey demonstrated the considerable heterogeneity in the practice of PD among surgeons in India. Considering the complex nature of this procedure with numerous preoperative, intraoperative, and postoperative implications, evident from the questionnaire we used, one can expect considerable heterogeneity in its practice.

One notable demographic finding in the survey was that all respondents were male, concurrent with the poor female representation in general surgery, especially hepatobiliary pancreatic surgery, globally [6]. Women's representation in India is not well documented. It is well known that perioperative outcomes of complex procedures such as PD depend on procedure volume [2,7]. The definition of high volume varies among literature, though an annual volume of more than 20 is generally considered high volume [7,8]. As per this definition, less than 30% of surgeons in our survey were high-volume service providers. This points to the need to centralize complex cancer surgeries such as PD in India.

Preoperative biliary drainage in pancreatic cancer in the absence of cholangitis or any other factors precluding a timely surgery is generally not recommended, as evident from current literature [9]. But drainage based on serum bilirubin level is still controversial [10,11]. Our survey results reflect this controversy, with 56.4% of the surgeons believing high serum bilirubin level is an indication of biliary drainage

Various professional bodies have attempted to categorize pancreatic cancer into resectable, borderline resectable, and unresectable/locally advanced pancreatic cancer with slight variations and recommended management strategies for each [12-14]. It is worth noticing that nearly all the survey participants (95.5%) concur with them and practice accordingly. It also means that efforts to standardize the management of pancreatic cancer are becoming fruitful.

Even though early attempts for minimally invasive pancreas surgery kickstarted in the 1990s, the adoption of the same was very slow owing to apparent factors such as the complexity of the procedure, lengthy learning curve, etc. The adoption is still very low when it comes to PD, with a 2019 American registry-based study showing that just more than 15% of the PDs were performed minimally invasive [15]. In India also, adoption seems to be low as per our survey, with only 8.2% performing >10% PD and 2.8% performing >50% PD minimally invasive.

Classical PD and PPPD were equivalent in morbidity and oncological terms, the same as PPPD and PRPD, based on meta-analyses of randomized controlled trials (RCTs), the highest level of evidence [16,17]. Pancreatic surgeons in India are doing all three proximal GI tract division methods, with 40% each for classical PD and PRPD and 20% preferring PPPD. Contrary to the current evidence, 40% of surgeons believed PPPD increases delayed gastric emptying. Data from retrospective studies might have influenced their decision [18]. There is some evidence that preservation of the stomach improves nutritional status after a PD [19], and one-fourth of our survey participants believed so.

As per the International Study Group on Pancreatic Surgery (ISGPS) consensus statement, standard lymphadenectomy for pancreatoduodenectomy should strive to resect lymph node stations 5, 6, 8a, 12b1, 12b2, 12c, 13a, 13b, 14a, 14b, 17a, and 17b [20]. From our survey, it is evident that most of the surgeons were not following the recommendation from the expert panel, which emphasize further the need for centralization and standardization of the procedure.

PJ and PG do not differ in the rate of POPF and overall complication rates, as evident from multiple well-conducted RCTs and meta-analyses of RCTs [21]. Still, the rate of post-pancreatectomy hemorrhage is slightly higher with PG. This might be the reason that most surgeons prefer PJ over PG. The inheritance of the training might have also influenced this preference for PJ over PG.

Invagination and duct-to-mucosa techniques do not differ in terms of POPF rates as per the latest literature [22,23]. Still, there is some evidence that in high-risk cases, invagination techniques help [22]. Among Indian surgeons, more than 70% use duct-to-mucosa techniques, while one-fourth use invagination techniques. When it comes to using trans-anastomotic stents, the quality of evidence comparing the use of stents versus no stents in terms of the development of POPF was low, precluding the derivation of any valid conclusions; the same applies to the evidence comparing the use of internal versus external stents [24]. Only a third of the survey participants use stents routinely.

The use of prophylactic octreotide is generally found to be not helpful in preventing POPF [25]. In our survey, 20% of surgeons used it routinely, and more than 60% used it selectively in high-risk pancreas such as soft pancreas, small pancreatic duct, etc. POPF is still the Achilles heel in PD, and the fear of it might be pushing surgeons to use it based on belief rather than evidence.

Currently, there is no high-quality evidence comparing biliary-enteric anastomosis techniques. Comparative data on the different methods of hepaticojejunostomy is available only in the context of liver transplants and few retrospective studies. These studies suggest that an interruptedly sutured hepaticojejunostomy is associated with a higher leakage rate [26]. Our survey participants put their faith in interruptedly placed sutures, with more than half using it routinely and only 10% using continuous sutures routinely.

The usage of a drain in PD was a controversial subject till 2014, when an RCT published data favoring the usage of drains, stating that avoiding drains increases morbidity and mortality [27]. In our survey, all the participants believed in routine drainage; the difference in opinion was only on the number of drains

Even though many studies bring the complications of feeding access to light [28], nearly all survey participants believed that feeding access is a must in the form of FJ or NJ in PD, with more than half relying on a jejunostomy tube. Early oral feeding is found to be safe in pancreatoduodenectomy patients [29]. In our survey, only a third of the surgeons started early oral feeding.

ISGPS defines POPF as a drain output of any measurable fluid volume with an amylase level greater than three times the upper limit of institutional normal serum amylase activity on or after postoperative day three, associated with a clinically relevant development/condition related directly to the postoperative pancreatic fistula [30]. It has graded POPF based on clinical severity. Following the definition, nearly 90% of the survey participants checked drain fluid amylase levels on day three and after that at different time points per availability of resources and personal preference.

In the literature review, we identified three surveys assessing PD practice [3-5]. Two surveys were country based; the United Kingdom (UK) and Brazil, and the third one was a worldwide attempt. The results of the worldwide survey and ours mirror each other in many aspects [4]. Globally, only 35% of surgeons meet the threshold for a high-volume surgeon. In our case, it was slightly less. PJ is the preferred reconstructive method globally (88%); the same as we found in our survey. Trans-anastomotic stents are not so popular globally; only 16% use them always and 54% use them selectively. Similarly, a third of our participants used stents routinely and selectively. Participants from both surveys do not prefer the routine use of prophylactic octreotide. But in a few aspects, Indian practice differed from global practice. All Indian surgeons preferred to do routine drainage, whereas globally, routine intraperitoneal drainage was reported by 59.2% of surgeons. Compared to the global survey, our survey was more exhaustive, and contained all the aspects of PD starting from preoperative evaluation to postoperative care

The survey from the UK had 57 participants [3]. Similar to our survey results and the worldwide survey, high-volume surgeons constituted less than a third of the participants in the UK (23% performing more than 15 PDs). Routine preoperative biliary drainage was done by half (51%) of the surgeons in the UK. However, we need to remember that the survey was published in 2007, much before the evidence against biliary drainage was presented. In the UK also, PJ was the preferred drainage method (95%). Unlike Indian surgeons, they did not prefer placing feeding access intraoperative with only 36% placing a jejunostomy tube.

The Brazilian survey had 51 participants [5]. In Brazil too, high-volume surgeons were only 28%. Adoption of minimal invasive PD was similar to the Indian experience with 65% of surgeons performing open PD (62% in our survey). The practice of transection level of the proximal GI tract was also similar with 46% preferring classical PD (40% in our survey). Similar to UK and Indian surgeons, they preferred PJ (95%). Routine use of octreotide was also not preferred (11%).

Despite the valuable insights gained through questionnaire surveys, it is crucial to recognize their potential for bias. Incomplete responses, idealized answers from clinicians, preferential responses from participants, and limited statistical power can all skew the results. This is particularly true in a sprawling country like India when the pool of surveyed surgeons is limited in size, leading to additional limitations in the study.

As far as we are aware, this survey marks a pioneering effort to explore the practices surrounding PD in India, a country home to one-fifth of the world's population. A deeper understanding of the practice of complex surgical procedures like PD will be instrumental in standardizing surgical procedures, a pressing need in today's world.

## Conclusions

The survey results provide valuable insights into the significant disparities observed in the surgical practices of PD among surgeons in India. This heterogeneity is not limited to a particular aspect of the procedure but is apparent across all stages, including evaluation, surgical technique, and postoperative management. The wide range of approaches and preferences among surgeons highlights the urgent need for standardization in order to optimize patient outcomes. Standardization can facilitate a more consistent favorable patient outcomes.

## Appendices

Pancreaticoduodenectomy survey

1. Email

2. Number of years after obtaining the highest degree (Mark only one ).

A) < 5

B) 6-10

C) 11-20

D) >20

3. Check all those which apply with regard to the surgical degrees obtained (Tick all that apply).

A) MS/DNB Surgery

B) MCh/DNB Surgical Oncology

C) MCh/DNB Surgical Gastroenterology/GI Surgery/HPB Surgery

D) Fellowship in GI/HPB surgery

E) Other:

4.  Your Gender (Mark only one).

A) Female

B) Male

C) Prefer not to say

D) Other:

5. Your Age (in completed years).

6. Average number of pancreaticoduodenectomies per year (either performed or supervised directly while being in the operating room)

(Mark only one).

A) <5 years

B) 5-15 years

C) 15-30 years

D) >30 years

7. The proportion of patients (approx.) in your practice referred to you after CBD stenting (Mark only one).

A) <25%

B) 26-50%

C) 51-75%

D) >75%

8. At what bilirubin cut-off level would you consider biliary stenting (in a good-risk patient and absence of other indications for stenting) (Mark only one).

A) >15 g/dl

B) >20 g/dl

C) >25 g/dl

D) I don’t believe in stenting uncomplicated cases irrespective of bilirubin level

E) Other:

9. A patient comes to you with serum albumin 2.3 g/dl; your usual policy before Whipple’s is (Mark only one).

A) Preoperative nutrition to bring up to a target albumin level.

B) Preoperative nutrition to see an increasing trend of albumin and not chase a specific value

C) Add human albumin infusion in addition to diet to quickly raise the albumin to desired level

10. In a patient with pancreatic head/ periampullary carcinoma, In which of the following conditions suspected on preoperative CECT scan, you will consider upfront surgery as a curative option (check all that apply)

A) Venous abutment

B) Infiltration of SMV/PV, but no collateral/cavernoma formation tumor thrombus in vein

C) Arterial (SMA/CHA) abutment

11. Approach to surgery (Mark only one).

A) Always open

B) Invariably open but have tried laparoscopy once or twice

C) Majority open, very selective minimal access (<10%)

D) Mostly open, minimal access between 10-50%

E) >50 % minimal access (robotic/laparoscopic)

12. Preferred level of proximal GI tract division (Mark only one).

A) Classical PD with resecting distal 1/3rd stomach

B) Pylorus preserving PD

C) Pylorus resecting PD (stomach divided 1-2 cm proximal to pylorus)

13. Your views/philosophy regarding the level of proximal GI division (check all that apply).

A) More the extent of gastric resection better the lymphadenectomy

B) Pylorus preservation increases delayed gastric emptying

C) The extent of gastric resection parallels post-operative malnutrition

D) Pylorus preservation reduces bleeding and operating time

E) Other:

14. The extent of lymphadenectomy (check all that apply)

A) Standard lymph nodes which are removed with the specimen

B) Always remove CHA node

C) Complete HDL nodal tissue

D) I always like to add/sample para-aortic nodes

E) I always like to add/sample celiac nodes

15. Method of pancreatic transection (Mark only one)

A) Cold knife

B) Monopolar cautery

C) Energy devices (e.g. Harmonic scalpel) for majority and sharp incision for duct

D) Other

16. Preferred organ for draining pancreatic remnant (Mark only one).

A) Invariably pancreatojejunostomy

B) Invariably pancreaticogastrostomy

C) Tailored (either PG or PJ) as per pancreatic texture/ duct size, duct location, etc.

D) Pancreaticojejunostomy using isolated roux limb

17. Preferred technique of pancreatic digestive anastomosis (Mark only one).

A) Invagination variants (opening of jejunum/stomach corresponding to the size of the pancreatic stump)

B) Duct to mucosa (jejunal/gastric opening corresponding to PD size)

C) Binding

D) Other:                                                                        

18. Do you use magnification (Surgical loupe/ microscope) during anastomosis? (Mark only one).

A) Always

B) Never

B) Selectively- for small ducts

C) I use whenever available (I do not have personal loupes)

19. Do you stent pancreatic anastomosis (Mark only one)

A) Routine

B) Never

C) Selective

20. How do you place stents in pancreatic anastomosis? (Mark only one).

A) Internal

B) External

C) Don't use at all

21. Your preferred technique for biliary-enteric anastomosis (Mark only one).

A) Continuous

B) Interrupted

C) Combination of the above in each patient (e.g. posterior continuous and anterior interrupted or vice versa)

D) Continuous for large diameter duct and interrupted/ combination for small diameter duct

22. Use of perioperative octreotide or its analog (Mark only one).

A) Routinely in all cases

B) Never        

C) Selectively in small duct and soft pancreas

D) Other

23. Timing of first dose of octreotide?

A) At induction of anesthesia

B) Just before transecting pancreas

C) just before starting pancreatic anastomosis

D) During or After completing anastomosis

E) only postoperatively if drain fluid amylase is high

F) Other:

24. How long do you continue octreotide? (Mark only one).

A) Single dose

B) Upto three days after surgery

C) At least 5-7 days after surgery

D) Till drain fluid amylase is normal

E) Other:                                                                        

25. The usual duration of surgery (skin to skin) in uneventful cases (standard resection, no vascular resection, standard lymphadenectomy)

(Mark only one).

A) <5 hours

B) 5-8 hours

C) >  8 hours

D) Other

26. Your practice regarding intraabdominal drain after PD (Mark only one)

A) I don't believe in routine drainage

B) Skip occasionally

C) Mostly keep one drain, occasionally two

D) Mostly keep two drains, occasionally one

E) Invariably two

F) Other:

27. Your practice regarding feeding access after Whipple's (Mark only one) .

A) Feeding jejunostomy (FJ) invariably

B) Nasojejunal tube (NJ) invariably

C) NJ in low risk and FJ in perceived high risk for pancreatic fistula

D) None routinely and NJ if required postoperatively

E) none with parenteral nutrition backup if needed postoperative

28. How long do you keep the patient NPO in the absence of delayed gastric emptying (Mark only one).

A) I try to feed ASAP, definitely within 48 hours

B) Keep fasting for at least 48 hours

C) After the return of bowel sounds on auscultation

D) After the passage of flatus/bowel movement

E) Feed when the patient is hungry

29. When do you start NJ/FJ feeds after surgery (Mark only one).

A) Definitely within 24 hours

B) Only after 24 hours

C) After the return of bowel sounds

D) Only use it if necessary (I prefer oral feeds)

E) I don't put FJ/NJ tube

30. Choice of postoperative fluid (check all that apply)

A) NS

B) RL

C) D5

D) 1/2 NS

E) Albumin

F) Colloids

G) Other:

31. What is your protocol for measuring drain fluid amylase (DFA) levels (outside clinical study/ trial environment) (Mark only one).

A) POD 3 only

B) POD 3 and just before removal of drains

C) POD 3 and then on alternate days

D) Only if clinically indicated

E) I don't check at all

32. What is the most important consideration for you before removing the intrabdominal drain kept during PD? (Mark only one oval per row)

Amylase     Volume levels   Character of effluent 

Most important

Moderately important

Least important

33. For how many days of postoperative hospital stay do you counsel your patients based upon your past experience (in uncomplicated cases after PD) (Mark only one).

A) 5-7 days

B) 8-10 days

C) 11-15 days

D) Other:
